# Palliative care for people with dementia in the terminal phase: a mixed-methods qualitative study to inform service development

**DOI:** 10.1186/s12904-017-0201-4

**Published:** 2017-04-28

**Authors:** Jenny T. van der Steen, Natashe Lemos Dekker, Marie-José H. E. Gijsberts, Laura H. Vermeulen, Margje M. Mahler, B. Anne-Mei The

**Affiliations:** 1 Department of Public Health and Primary Care, Leiden University Medical Center, Hippocratespad 21, Gebouw 3, P.O. Box 9600, 2300 RC Leiden, The Netherlands; 2 Department of Primary and Community Care (117 ELG), Radboud university medical center, Geert Grooteplein Noord 21, P.O. Box 9101, 6500 HB Nijmegen, The Netherlands; 3 Amsterdam Institute for Social Science Research (AISSR), University of Amsterdam, Nieuwe Achtergracht 166, 1018 WV Amsterdam, The Netherlands; 4 EMGO Institute for Health and Care Research, Department of Public and Occupational Health, VU University Medical Center, van der Boechorststraat 7, 1081 BT Amsterdam, The Netherlands; 5 Stichting Kalorama, Department of Psychology, Nieuwe Holleweg 12, Beek-Ubbergen and Kennis door Verbinding, Nieuwe Mollenhutseweg 15, 6533 HB Nijmegen, The Netherlands; 6 Tao of Care, Zwanenburgwal 206, 1011 JH Amsterdam, The Netherlands

**Keywords:** Dementia, End of life, Health services, Hospice care, Palliative care, Program development

## Abstract

**Background:**

When entering the dying phase, the nature of physical, psychosocial and spiritual care needs of people with dementia and their families may change. Our objective was to understand what needs to be in place to develop optimal palliative care services for the terminal phase in the face of a small evidence base.

**Methods:**

In 2015–2016, we performed a mixed-methods qualitative study in which we (1) analysed the domains and recommendations from the European Association for Palliative Care (EAPC) dementia white paper and identified those with particular relevance for the terminal phase; (2) performed a series of focus group discussions with Dutch family caregivers of people with dementia in variable stages; (3) conducted interviews with experts involved in 15 special forms of terminal care for people with dementia in five countries. The terminal phase was defined as dying but because of the difficulty predicting it, we included advanced dementia. We initially analysed the three parts separately, followed by an integrated analysis of (1)-(3) to inform service development.

**Results:**

(1) The EAPC domain of “avoiding overly aggressive, burdensome, or futile treatment” was regarded of particular relevance in the terminal phase, along with a number of recommendations that refer to providing of comfort. (2) Families preferred continuity in care and living arrangements. Despite a recognition that this was a time when they had complex support needs, they found it difficult to accept involvement of a large team of unfamiliar (professional) caregivers. Mostly, terminal care was preferred at the place of residence. (3) The expert interviews identified preferred, successful models in which a representative of a well-trained team has the time, authority and necessary expertise to provide care and education of staff and family to where people are and which ensure continuity of relationships with and around the patient.

**Conclusion:**

A mobile team that specializes in palliative care in dementia and supports professional and family caregivers is a promising model. Compared to transfer to a hospice in the last weeks or days, it has the potential to address the priorities of families and patients for continuity of care, relationships and specialist expertise.

## Background

There is no cure for dementia, and in our aging societies, people increasingly die with or from dementia or from complications that frequently develop as part of the dementia [1–3]. Dementia can be perceived as a chronic-progressive yet terminal disease which involves complex needs [4]. In some respects, such as a large symptom burden, it resembles cancer [4, 5]. However, unlike a cancer palliative care trajectory, people in the advanced stage of dementia may still live for a number of years, while some never reach an easily recognizable terminal stage [6–8]. The course of the disease is more difficult to predict than the course of cancer as it also depends on when, for example, pneumonia or food and fluid intake problems develop, which are not limited to the advanced stage [6]. The inevitable cognitive decline with dementia, which results in patients not always being aware of the disease also complicates how patients are assessed and involved in care-related decision making. This means that the involvement of family members warrants a particular focus from and significance for professional caregivers. Family members are both caregivers and proxy decision maker in need of extra support and information; for example, to anticipate future events and understand the terminal nature of the disease [3, 9–11].

Along with suffering from anticipatory grief (pre-grief), they also need to deal with aspects of care that are not so evident for other patients who are dying, e.g., due to behaviours that challenge, and patient distress and disorientation. When this is compared with the last year of life of people without dementia residing in the community, these family carers report having fewer positive caregiving experiences [12].

Due to the terminal nature of dementia and complex needs, people with dementia and their families can and should benefit from access to palliative care. The European Association for Palliative Care (EAPC) dementia white paper asserts that although the palliative approach may be applied from the time of diagnosis, it becomes increasingly important for people with more severe dementia, for those who are at the point when quality of life is a more relevant care goal than the length of life [4]. The experts in the Delphi study that resulted in the EAPC dementia white paper, however, did not agree on the stages of dementia for which palliative care was (most) important. Further, the EAPC white paper includes 57 recommendations for practice, policy and research. Nevertheless, with few good practices and intervention studies available, it is still unclear how to shape palliative care in dementia in practice and what effective care would look like at different points in the dementia trajectory. This includes the terminal phase-the last days or weeks of life, or at most, a few months. People with advanced dementia but also people with dementia in earlier stages may be in the terminal phase [6]. Care in the terminal phase may require a specific approach, e.g. in the communication with relatives, and there is less time to evaluate effects of treatment, while some treatments may be withdrawn or withheld.

The question that guided our research was how to develop the best practice for care for people in the terminal phase of dementia (so, at the end of life, but not necessarily with advanced dementia) for implementation in the Netherlands. We adopted an evidence-based approach that drew on empirical evidence, experiential knowledge and case studies of service provision.

## Methods

This was a mixed-method study involving evidence review and primary research with key stakeholders. We (1) identified evidence about needs of people with dementia in the terminal phase; (2) performed focus group discussions with Dutch family caregivers of people with dementia about possible needs in the terminal phase; (3) conducted interviews about innovative, specialized forms of terminal care for people with dementia in five countries.

### Evidence

There have been a number of descriptive studies about care at the end of life or in advanced dementia [5, 13]. However, there has been a dearth of literature on service development, apart from US initiatives in the 1980s [14, 15]. Because of the disappointing results of search strategies combining hospice/end of life/terminal care with dementia, we concluded that to learn more about service development and its facilitators and barriers, we needed to look for non-scientific reports, search for projects and health care services specialized in care for people with dementia in the terminal phase and consult with experts.

We used the EAPC white paper on palliative care in dementia as a recent resource that has both synthesized a diverse literature and achieved a consensus, based on the available evidence, among experts about best practice in palliative care in dementia [4]. First, three team members independently identified recommendations that were most or more relevant to the terminal phase (dying or advanced dementia). Agreement was reached through discussion. Next, to validate our prioritization, we asked seven of the twelve authors of the EAPC white paper to review our selection of recommendations that we found of particular importance in the terminal phase. We selected involved authors with different clinical backgrounds and one author that represented a patient society. We ensured that at least one author from the six countries of residence of the twelve authors participated. We adapted in response to the comments and with divergent comments, we fed back comments from other EAPC white paper authors to resolve discrepancies in discussion where possible; our team agreed to the final version.

### Focus group discussions with family caregivers

Focus group discussions were conducted with two groups of family caregivers. Each group met three times between 1 September and 20 October 2015 with about 2 to 4 weeks in-between. We involved two groups to cover more variability in experiences in caregiving. In the first session, the participants reflected on the time around diagnoses, the second session focused on experiences since that time, and the last session in each group focused specifically on the topic of end-of-life care for people with dementia. Whereas end-of-life issues were addressed mostly in the third focus group session, end-of-life issues which came up in the first and second sessions, and a fourth and final feedback session in July 2016 were also taken into account for the current analysis.

#### Recruitment of participants

The participants knew each other as they were recruited via the support groups held at the day center their spouses frequented. Group 1 involved six caregivers of people with dementia living at home (three females, three males, aged 59–86), five of whom were spouses and one was daughter-in-law. Group 2 comprised four wives, aged 60–75. This group included one bereaved family member, two participants whose loved ones lived in a nursing home and who were preparing for transfer, and one participant whose loved one lived at home. Of the ten people with dementia they cared for, seven were diagnosed before age 65. At the time of the interview it had been between 1 and 11 years since they received their diagnosis. Whereas home care services are generally widely available in the Netherlands, with the exception of one caregiver, none of the participants had received help from home care services at the time of the first interview round. Some would occasionally receive help from their family and friends. All but two of the people with dementia would frequent a day centre for at least 1 day a week. All caregivers were supported by dementia case-managers, and five had received psychological help to support them in the emotional challenges posed by their loved-one’s illness trajectories and their demanding caring roles.

#### Convening of the focus group sessions

The discussions were led by the person who also usually moderated the support groups which ensured that participants were supported and were comfortable discussing sensitive topics. Two to three authors attended with one of them observing group interaction and the others asking questions. The discussions were transcribed verbatim including description of atmosphere and context.

#### Analyses

Coding of content and an inductive thematic analysis was performed by two anthropologists (LV and NLD). Afterwards, a draft of this article’s section on the focus group discussions was shared with the participants in the fourth and final session, as a member check. All participants agreed to and confirmed the section’s contents. Additional material on the recent death of one of the participants’ loved one came up during the feedback session and was integrated into the analysis. The identifying of themes in focus group and experts’ interviews was performed independently, while fine tuning and interpretation was an overall team effort.

### Expert interviews

Interviews with experts who were involved in terminal care services in dementia were held in 2015 and up to March 2016. The interviews were with individual experts, or, in some cases, the interviewees felt a group interview to be more informative and they asked colleagues to join.

#### Recruitment of experts and identifying relevant initiatives

Through our national and international networks in dementia care and palliative care, and later also through interviewees’ suggestions, we identified experts (project leaders, key staff members). The experts were developing or had developed and practiced a special form of palliative care services for people with dementia at the end of life (terminal phase). The target group’s experience also included services aimed at people with advanced dementia so as to learn from experiences in this related group with overlapping needs, also addressing the well-known problems with prognostication so that people may live longer or shorter than expected. For the same reason, we also included services providing care to people with dementia who were not expected to live longer than 6 months.

#### Convening of the interviews

The interviews started by asking the experts how they identified the target group and developed their service. As the interviews progressed, key issues in relation to terminal care for people with dementia were identified, and we developed an interview guide (Table 1). The guide listed important barriers to optimal use of the services that emerged from the first series of interviews. This was an iterative process, and when appropriate we went back and asked additional questions to those who had been interviewed in the first phase of data collection.

**Table 1:** Interview guide

Opening	
• Can you tell about your service? (cues: hands-on or consultation, to what extent is it multidisciplinary care, covering palliative care domains such as any spiritual care), what is different compared to “usual care”? (e.g., any use of assessment tools, more staff, peaceful environment, staff training). How did it start, whose idea was it?	
The transition and possible related barriers	
• Selection of patients, eligibility for the program • How is a transfer to another place, or a referral to palliative care that is provided where people are, being experienced by patient, family and health care professionals? • What is the public image of this type of care (also as compared to, e.g., nursing home care) and how do you call this type of care in encounters with family and patient?	
Pros and cons	
• What works well for these people with dementia, and why? • What could be improved, how so?	

The interviewers were team members (JTS, NLD, MJG; one or two interviewers for each interview). In one case, one of the interviewed experts accompanied us and participated in another expert interview to learn about that specific initiative. We suggested to cover visual communication (e.g. through Skype), but the preferred medium was based on the preference of interviewees. Site visits were conducted by invitation, when feasible. All interviews were audiotaped (except for one not in full due to failure of the recording device).

#### Analyses

Thematic analysis was conducted concurrently with the interviews to ensure that key ideas or themes could be explored in more depth in subsequent interviews and revisited if needed with those who had already participated. This involved familiarization with the data, by the researchers listening to the recorded interviews, a 2- to 5-page summary with annotated notes of every interview that included the team’s reflections on important issues for the development of new services. The ongoing analyses during data collection thus iteratively led to identification of the most important elements, and it also resulted in additions to the interview guide and informed selection of further candidates for interviews. In most cases, new questions emerged, and we fed back to the interviewees the summaries along with questions and reflections from the team. The responses and suggestions were used to improve the summaries and reflective notes.

While we initially analysed the three parts separately, this was followed by an integrated analysis of (1)-(3) to inform service development. We related the perceptions and experiences of the experts and family caregivers to the most relevant recommendations in the terminal phase selected from the EAPC dementia white paper to recommend on service development.

### Context of usual care in the Netherlands

The context is important to interpret the results in terms of recommendations for implementation in the Netherlands. Here, most patients with dementia live at home, but almost all die in institutional long-term care settings [16, 17]. Certified elderly care physicians coordinate multidisciplinary teams in nursing homes. In residential homes, the general practitioner provides most generalist palliative care but elderly care physicians may be involved in a consultancy role.

Previous work had found that comfort at the end of life in people with dementia was worse in residential settings compared to comfort in nursing homes [18]. Elderly care physicians receive some training in a palliative care approach. However, specialists in palliative care are rarely consulted [19]. Elderly care physicians usually initiate advance care planning upon admission to the nursing home [20, 21] but the process varies (e.g., assess global care goal only or also discuss treatment options) and patient advance directives are rare. The physicians are gatekeepers which results in few hospitalizations, and withholding of futile curative care is a common practice [22]. Euthanasia in dementia is widely debated but rarely performed because, in principle, the patient needs to be able to understand the situation and confirm the request. The care workforce often does not have any systematic training or education in end-of-life care and the facilities rely on nurses with minimal qualifications supported by unqualified care staff (e.g. most homes do not employ any BSc-level nurse, but only nurse aids and nurse assistants; nurses with comparable training and responsibilities as licensed practice nurses).

### Ethics and confidentiality

All participants provided consent for recording of the discussions and analyses of coded transcripts for scientific purposes. The focus group interviews were approved by the Amsterdam Institute for Social Science Research (AISSR) Ethical Advisory Board and the Social & Behavioural Sciences Faculty Committee of the University of Amsterdam (2014-AISSR-3805). To maintain expert confidentiality, we do not refer to papers describing the initiatives which were available in some cases.

## Results

### Evidence from EAPC recommendations

Table 2 shows 22 of the 57 recommendations from the EAPC dementia white paper that we identified as of particular importance for the terminal phase (dying or advanced dementia). Two of the 22 were specific to this phase only in part. The other 25 recommendations were found either equally important, or less important for the terminal phase.

**Table 2:** The 22 of 57 EAPC recommendations of special importance in late stages: severe dementia and around dying

Domain 1. Applicability of palliative care (1/4 recommendations)
1.2 Improving quality of life, maintaining function and maximizing comfort, which are also goals of palliative care, can be considered appropriate in dementia throughout the disease trajectory, with the emphasis on particular goals changing over time.^a^
Domain 2. Person-centred care, communication, and shared decision making (0/6 recommendations)
Domain 3. Setting care goals and advance planning (1/7 recommendations)
3.5 In more severe dementia and when death approaches, the patient’s best interest may be increasingly served with a primary goal of maximization of comfort.
Domain 4. Continuity of care (1/4 recommendations)
4.1 Care should be continuous; there should be no interruption even with transfer
Domain 5. Prognostication and timely recognition of dying (1/2 recommendations)
5.2 Prognostication in dementia is challenging and mortality cannot be predicted accurately. However, combining clinical judgement and tools for mortality predictions can provide an indication which may facilitate discussion of prognosis.
Domain 6. Avoiding overly aggressive, burdensome, or futile treatment (6/6 recommendations)
6.1 Transfer to the hospital and the associated risks and benefits should be considered prudently in relation to the care goals and taking into account also the stage of the dementia. 6.2 Medication for chronic conditions and comorbid diseases should be reviewed regularly in light of care goals, estimated life expectancy, and the effects and side effects of treatment. 6.3 Restraints should be avoided whenever possible. 6.4 Hydration, preferably subcutaneous, may be provided if appropriate, such as in case of infection; it is inappropriate in the dying phase (only moderate consensus). 6.5 Permanent enteral tube nutrition may not be beneficial and should as a rule be avoided in dementia; skilful hand feeding is preferred (only moderate consensus). 6.6 Antibiotics may be appropriate in treating infections with the goal of increasing comfort by alleviating the symptoms of infection. Life-prolonging effects need to be considered, especially in case of treatment decisions around pneumonia.
Domain 7. Optimal treatment of symptoms and providing comfort (4/6 recommendations)
7.1 A holistic approach to treatment of symptoms is paramount because symptoms occur frequently and may be interrelated, or expressed differently (e.g., when pain is expressed as agitation). 7.2 Distinguishing between sources of discomfort (e.g., pain or being cold) in severe dementia is facilitated by integrating views of more caregivers. 7.3 Tools to assess pain, discomfort and behaviour should be used for screening and monitoring of patients with moderate and severe dementia, evaluating effectiveness of interventions. 7.5 Nursing care is very important to ensure comfort in patients near death.
Domain 8. Psychosocial and spiritual support (2/4 recommendations)
8.3 Religious activities, such as rituals, songs, and services may help the patient because these may be recognized even in severe dementia. 8.4 For dying people, a comfortable environment is desirable.
Domain 9. Family care and involvement (3/8 recommendations, of which 1 only in part)
9.2 *(in part)* Families may need support throughout the trajectory, but especially upon diagnosis, when dealing with challenging behaviour, with health problems, with institutionalization, *with a major decline in health, and when death is near.* 9.7 Bereavement support should be offered. 9.8 Following the death of the patient, family members should be allowed adequate time to adjust after often a long period of caring for the patient.
Domain 10. Education of the health care team (0/2 recommendations)
Domain 11. Societal and ethical issues (3/8 recommendations, of which one only in part)
11.3 Collaboration between dementia and palliative care should be promoted. 11.6 Economic and systemic incentives should encourage excellent end-of-life care for patients with dementia. 11.8 *(in part)* National strategies for dementia, for palliative care, *end-of-life care,* and for long-term care should each include palliative care for dementia patients. Similarly, policy making on palliative care and long-term care settings should attend to dementia.

^a^Note that the Figure which belongs to this recommendation visualizes increasing importance of maximization of comfort with more severe dementia

All 6 recommendations of domain 6, “Avoiding overly aggressive, burdensome, or futile treatment” were of particular relevance in the terminal phase, along with 7 recommendations that refer to providing of comfort (4 of 6 recommendations of domain 7, “Optimal treatment of symptoms and providing comfort” along with 3 recommendations from other domains: 1.2, 3.5, and 8.4). The other more important recommendations referred to continuity of care (4.1), prognostication (5.2), spiritual support (8.3), family support (9.2, 9.7, 9.8) and societal and ethical issues (11.3, 11.6, 11.8). The domains of “Person-centred care, communication, and shared decision” (domain 2) and “Education of the health care team” (domain 10) were deemed *important but not more so* in the terminal phase than in any other phase. Whether or not recommendations 4.1 (on continuity of care) and 7.1 (a holistic approach to treatment of symptoms) were more relevant for the terminal phase was a subject of discussions. We finally added these as late transfers may risk the loss of personhood (no time to build up relationships, understand the person’s biography and their preferences and priorities). It was thought that even if in earlier phases the risk of confusion may be larger, the complexity of symptoms in the terminal phase still meant that continuity, and a holistic rather than mostly medical, and a person centred approach were even more relevant.

### Family views from focus group discussions

The focus group discussions about the families’ experiences with caregiving and preferences for end-of-life care indicated that they were struggling with every-day care. They spontaneously brought up the possibility of euthanasia and welcoming death as a release for their relative. Probing resulted in broader discussions and we identified two main themes that captured what family members saw as important when providing terminal care: communication and familiarity. Both themes related to a desire for continuity in several respects, to continue on familiar grounds as long as possible.

#### Communication

Many of the carers had become socially isolated having lost friendships and close connections with family and friends in the period of caring for their loved-ones. Consequently, continuity in the communication with professional and family caregivers was identified as important by participants both for their support and for good care at home and in a nursing home. For those caring at home, almost all expressed a strong appreciation of long-standing supportive relationships with one or more persons, their GP, a volunteer, a psychologist, or case-manager, either of whom appeared to be a trusted person and companion throughout the caring process. In both groups fears were being expressed about the prospect of their loved one moving to a nursing home and their ability to maintain involvement in their relative’s care. Would a euthanasia request still be granted by the new elderly care physician? Would their own expertise in caring for their loved one be taken into account by the new care staff? And when talking about care in the nursing home, all expressed their expectation or appreciations of being consulted, involved in and guided throughout the care process by nursing staff and physicians. The following quote illustrates one woman’s experiences from group 2 in her husband’s last days of life, and the importance of good communication with the nursing home’s care staff:

“*What I think is very important is that in the last stage there is good communication between yourself and the nursing staff. That you know exactly, what is going on? And that you also ask: could we do it differently, could we do it this way, that way? What can I actually do and what should I definitely not do? And that you are being guided in that process*.”

Also in group 2, a case was discussed in which a family’s preference to withhold antibiotics was ignored. This caused ambivalent feelings of both relief and frustration about the patient surviving the infection (probable pneumonia). Others shared despair about not being allowed to participate in a multidisciplinary team meetings concerning planning of care of their love one. The fact that different physicians were on duty in nursing homes (some only 2 days a week, or changing staff), was often seen as a cause of miscommunication and deviations from end-of-life care planning’s as previously agreed on. Also more generally, carers did not like the fact they had to explain their family's end-of-life preferences repeatedly; each time a new elderly care physician was on duty (elderly care physicians are on the staff in Dutch nursing homes).

#### Familiarity

Familiarity and the importance of continuity in caring relationships was stressed as important and particularly emphasized as preferable to the situation of different people coming in all the time:

*“And absolutely no different people taking turns in caring for him. That is confusing. You just cannot do that. I would never want that to happen.”*

All focus group participants believed home to be the best place for end-of-life care. It was agreed that people with dementia should not be moved to a hospice when they are dying, although many admitted they doubted whether they would be able to keep caring for their loved ones in their own homes alone, with no additional support, until the very end. These quotes shows fears in group 2 that the transfer could exacerbate rather than improve the person with dementia’s orientation and sense of place:

“*and then there is no need for a hospice, since a hospice would mean another transfer*” [in addition to transfer to the nursing home]. Another, in response: “*When you are already so confused. And since this already takes its natural course. R [her husband] was there for seventeen months and he just went out like a candle*.” Yet another, with a home-living husband, in response: “*we have already decided, we are not going to move. No moving, no matter what. No way. As long as he is there. Because this is familiar*! *Or if somebody has reached his final days, then he should not be taken from his environment. This does happen, it happens often in nursing homes. I know that. That is terribly confusing. You just cannot do that.”*

An approach that achieved a sense of the familiar, with the support of on-site care was described by one bereaved family member; she referred to the last weeks in the nursing home (small-scale living) as “*a beautiful time*” with her husband in his own room, surrounded by his own “belongings” and under his own bed linen. She recommended other participants to bring as much familiar things as possible to the nursing home, to make it a real home. In her narrative of that period however, the home metaphor was prevalent in a way moving beyond the importance of physical surroundings. Specialized care was seen as unnecessary because existing relationships in the nursing home where her husband lived were sufficient to provide for warm and supportive care. She valued such strong bonds more than the idea of anything extra being done:

*But if you already have that bond established before, then nothing extra has to be done. Then it’s just a matter of trust. The nursing staff becomes - … and this may sound a bit overdone, but they become part of your family in a way.*

The others noted that such warm relationships were reflections of the opportunities to invest time in building a rapport and understanding with the nursing home staff such that they were included in the care and life of the nursing home. This extended to the daughter being able to stay there, and she concluded:

*“That was why it felt like home for me, and why it felt like home for my daughter, who also stayed there during the last couple of days.”*

Finally, the importance of continuity in caring relations and place was mentioned as extending beyond the moment of death itself. In the feedback session, a group 1 participant reflected on the recent death of one of the participant’s loved ones, and her own experiences with deaths in her husband’s nursing home. She recollected how a lack of communication about the death of former co-residents time and again caused anxiety among residents in the small scale living arrangement. She also mentioned how important it was to accommodate for a quiet and welcoming place where visitors could come by and pay their respects at the place of death. The (nursing) home after all was the deceased person’s “home.”

In summary, the focus groups suggested that–reflected in the themes communication and familiarity–a home-like atmosphere (and not always the person’s actual home) where family carers knew and trusted the staff involved in their relative’s care, and where they felt that their opinions would be listened to, were key issues when supporting someone at the terminal stage of life.

### Initiatives-expert interviews about special services

We held 17 interviews about 15 discrete palliative care initiatives run in 5 countries. The interviews lasted between 30 and 84 min (median: 48). Four were face-to-face, 4 via Skype and 9 were telephone interviews, and 2 telephone interview were followed by face-to-face group interviews on site in the Netherlands. Four were led by two interviewers, and 5 were with several (2 to 5) interviewees. In total, we interviewed 25 experts, 4 of whom were male; 11 physicians, 10 nurses, 1 social worker, and 3 project leaders not practicing as clinicians (currently).

#### The fifteen initiatives

Table 3 summarizes characteristics of the 15 initiatives in 5 countries. Six were from the Netherlands. Most initiatives in other countries covered outreaching or consultation to the community setting or the nursing home, mostly from specialist palliative care services with a focus on dementia. However, only one Dutch service comprised outreach of volunteers from the hospice to nursing homes. The initiatives varied considerably with respect to eligibility criteria, form, phase of development, and origin, with 8 originating from within a hospice or non-disease specific palliative care services and 7 were undertaken from dementia or institutional long-term care, including geriatrics.

**Table 3:** Characteristics of the 15 initiatives (17 interviews)

Characteristic	Number of initiatives
Country	
Netherlands	6
UK	3
US	3
Flanders	2
Israel	1
Target population of the initiative/enrollment criteria	
dying or life expectancy of at most 6 months	8
advanced dementia (and continued until death)	3
both	2
earlier possible (and continued until death)	2
Context/form	
consultancy, outreach, community	5
special nursing home department	4
hospice (institutional, inpatient)	2
combined hospice, and consultancy, outreach, community	2
nursing home center of excellence	1
special nursing home program	1
Phase of the initiative	
implemented	7
pilot phase	3
failure/changed/no longer exists	3
under development	2
Any formal description (such as a family brochure, scientific article)	
available	10
not (yet) available	5
Origin of the initiative/lead	
hospice/palliative care for other diseases	8
dementia/institutional long-term care	7

Tables 4 and 5 describe two services which we selected to illustrate the breadth of the initiatives. The Dutch bottom-up initiative in Table 4 attempted to recruit patients with advanced dementia for a hospice unit in the nursing home which was also used as a general hospice for people with no dementia in the last months of life. The main barrier to uptake of this service was family and nursing staff unwilling to move the patient from the familiar (nursing) home environment (“*We had to pry people loose, almost impossible to keep that up*”). For example, family replied, “*my loved one has resided in this unit for so long now and we know the staff: why at all move*?” This resonates with the focus groups’ findings. The unit subsequently closed and staff now employ a palliative approach for the residents who remain on the regular nursing home units.

**Table 4:** Example of an initiative of a palliative care unit for people with advanced dementia

Interviewee: physician	
Service description	
• Closed unit within a Dutch nursing home (part of a larger care organization with other nursing homes, hospice, and home care) • Combined 15-bed unit with 7 general palliative care beds and 8 beds for people with advanced dementia; all with their own rooms, a shared living room, and a seat for family • Bottom up initiative from a manager of small-scale living in the nursing home who felt that some people did not benefit from the activities they offered in the busy living room • Compared to regular nursing homes, this unit additionally offered specialized nursing care called “care for people who are Powerless in Daily Living” (PDL).^a^ A specialized therapist, but also the physiotherapist and occupational therapist worked according to the PDL-principles. Further, specialized chairs and mattresses facilitated comfortable positions. The unit had a snoozelen-bath with possibility for very immobile patients to soak in warm water, listening to music, scenting relaxing scents and looking at special lights. Additional staff and volunteers helped with feeding • Use of pain assessment tools specific for dementia and other tools such as for delirium, but no written protocols • Staff internal training in use of the tools and in palliative care more generally by a palliative nurse practitioner • Funded extra staff via the more generous insurance budgets for the general palliative care beds	
Admission criteria and patient recruitment	
• Admission with advanced dementia from psychogeriatric (dementia) units elsewhere in the nursing home, from home and hospital. People were usually ADL-dependent and communication was very limited, but the main criterion was that they did not benefit from being part of a group. No other diagnosis was required • Widely advertised (family brochure, admission office, general practice, hospital, talk to colleagues personally, consulted expert in communication). However, attempts to recruit patients with advanced dementia failed mainly because family and nursing staff were not willing to move the patient from a familiar environment (including familiar staff relationships) or did not recognize the person with advanced dementia not being comfortable there. Often it was not possible to convince them of benefits of the specialized care on the special unit	
Lessons learnt and shared in the interview	
• Beds remained empty because of the resistance to transfer at the end of life, which resulted in a lack of insurance funding and therefore the unit closed (had been in use for 2 years, 2013–2015) • Families of patients who did move to the unit were extremely satisfied with the care • People with advanced dementia do not fit well in a regular psychogeriatric nursing home department, but also do not fit well in a regular hospice. These people can benefit of a special approach or program • Better start with only a few beds and a team approach to recruit patients and show people the benefits of this approach. Alternatively, not transfer people and focus on bringing this care to where people are • What worked well is staff having expertise in both palliative and dementia care. The team spirit remained even if staff is working on a different unit now and PDL is provided on other units which is very comforting for people with contractures • People seemed to live long (2 to 3 years), perhaps even longer because of individual needs being met, staff responding to subtle changes in comfort • Physicians may feel that quality of life and comfort can be enhanced greatly through special nurse care giving	

^a^The PDL-technique aims to bring optimal relaxation and comfort during washing and clothing, lying in bed and feeding. It comprises slow care in connection with the resident and using different ways of sensory stimulation: touch, scents, music, and snoozelen activities. Clothing is adapted to the stiffness and impaired moving ability of arms and legs. Sometimes supportive pain medication is used [43]

**Table 5:** Example of a mobile palliative care team with staff specialized in nursing homes (where many people have dementia)

Interviewees: two nurses	
Service description	
• Palliative home care organization (“equipe”) in Flanders which also covers nursing homes • A nurse consultant is the coordinator who visits and supports patient, family and the regular (home) care staff. They do not take over the care (the GP remains in charge), but they support others (the environment) to provide palliative care. The nurse is part of the equipe’s multidisciplinary team but because of a pluralistic stance, no spiritual caregiver is directly attached to the team. Two nurses specialize in outreach to nursing homes, building relationships with staff; no hands-on care is provided, but for occasionally help with technically challenging nursing procedures • The initiative to also serve nursing homes was taken by this particular care organization decades ago and was reinforced by national policy afterwards • Compared to regular nursing home care: broad expertise in palliative and end-of-life care; compared to regular palliative care: experience in nursing homes • The team uses the nursing homes’ tools and protocols, if available, to not interfere with existing procedures • Staff is trained in palliative care (yearly refresher courses) and staff members specialized in nursing homes are trained with extra courses about dementia care and communication with dementia patients. Staff caring for patients (without or with dementia) at home do not receive special training in dementia, but if logistics allow, those specialized in nursing home care may attend a patient with dementia in the community • Funding by a fixed sum which means care is flexible and can be provided as needed for the individual patient no matter the number of visits	
Admission criteria and patient recruitment	
• Life expectancy at most 2 months but the service continues if patients outlive the 2-month funding limit • The criterion on life expectancy may reinforce misperceptions of palliative care as terminal care; however, relabelling of palliative care may not be the solution; rather, better explain palliative care as care that is different from a helpful, additional perspective rather than maximum care which may not be optimal care	
Lessons learnt and shared in the interview	
• Building personal relationships between the nurse coordinator and nursing home staff and the GP is very helpful, and there may be stages in the relationships. Initially, there has been resistance when the nurse became involved in a team of nurses and GP, with “their patient”. Experiencing how helpful the service is, may increase use even to the extent of overuse. At that point, it is important to encourage staff to learn to practice palliative care themselves • Still, the nurse consultant really has time to analyse the situation and develop a “helicopter view” which is difficult for staff in charge of providing the everyday hands-on care • Spiritual care is sometimes still neglected, also regarding needs of patient and family from different cultures or traditions • Searching for a consensus on care and treatment in the difficult situation of the patient unable to confer his or her preferences, is a major benefit of this service. Sometimes it takes time while the time window is limited; it may help if GPs have initiated advance care planning earlier and for this also increased public awareness is needed • The nurse also bridges practice between nursing homes as they confer tips to other nursing homes and link up homes that sometimes operate solitarily	

In contrast, the Flemish initiative in Table 5 was supported by national policy and palliative home care services specialized in nursing home residents who often have dementia. The consultation model overcame initial barriers of becoming involved late in the dying trajectory with an existing team of caregivers. In this account it was evident that there had been a process of learning of how to work with the care providers. In the early days GPs even “challenged the right to become involved with their patient.” We had to get “*a foot in the door*,” “*really push*.” Nowadays, the GPs may welcome a nurse who will “*do the dirty work for them*, *the heavy lifting*” because of reluctance among GPs to talk about death and dying (e.g., “ *to avoid an impression of wanting to get rid of a patient*”). This team had identified that educating professional and family caregivers was important so that they could be competent and comfortable providing care. The aim of the service was ultimately “*not being needed anymore*.” The service was flexible, with fixed funding, no fee for performance. Therefore, it did not matter if the consultant visited “*2 or 3 times a day or once a month*. ” “*Reputation*” and “*ethos*” were deemed strong enough to not increase costs unnecessarily. Searching for consensus on what should be done and what should not, however, may take time “*You can’t make grass grow by pulling it.”*

#### Themes

We identified three main themes from the interviews. These were familiarity, professionalism, and negative representations.

#### Familiarity

Familiarity related to relationships between professional caregivers, and between family and patient and professional caregivers. The experts found that an emphasis on the maintenance of relationships informed stated preferences for place of care, mostly to not transfer a patient and their family out of familiar surroundings. Further, often there was not a clear, urgent reason or emergency to do so at a particular point in time because there was no sudden deterioration.

Two experts had experienced difficulties in particular with transfer within their nursing homes to a specialized unit, as exemplified in Table 4. Nurses and family preferred to continue the care at the place of residence with the same caregivers over moving to a possibly more specialized, suitable yet unfamiliar environment and staff in the same building. Familiarity was also important for nurses. A possessiveness of the patient, along with not recognizing the potential benefits of transfer to a specialist unit (in the eyes of the interviewee), led them to prefer to not move the patient and risk losing connections with the patient and the family they knew so well. Similarly, with consultation models (Table 5) and also with involving volunteers for dying patients (not in Table), these people were unknown to the patient and family and sometimes or initially also new to the staff caring for the patient. This meant that before the “new people” could provide support they first had to clarify, defend and negotiate their role in order to convince the people around the patient of their expertise and added value of their involvement.

#### Professionalism

The experts invariably identified palliative care in the terminal phase as care that required a particular level of expertise. This included the importance of staff or volunteer attitudes and specialist expertise when caring for people with dementia at the end of life. They argued that nursing staff providing daily care, even when very dedicated, had educational needs. Of particular importance was the ability to interpret symptoms, communicate with people with dementia and notice changes in a person’s condition and act upon them. Some experts also referred to deficiencies in providing specialized palliative medical care by physicians (general practitioners but also elderly care physicians).

The experts consulted believed that some nursing staff members caring for people with dementia and hospice volunteers do not have the right attitude, interest and sensitive awareness to recognize special needs on how to bring comfort, to create a peaceful atmosphere, or to provide good nursing care at the end of life of people with dementia. Some mentioned that an optimal approach combines availability of medical care in the background with a frontline of social and human approach by dedicated nursing staff and volunteers. Without a combination of appropriate medical support and suitably qualified nursing staff and prepared volunteers, one expert observed that people with dementia are “*existing, not living*.” Of note, spiritual care was often not mentioned until prompted and it was not always addressed as systematically as the accounts of what usual care should entail. Finally, if prognosis was an enrolment criterion, identifying people who will benefit was complicated by difficulty predicting how long people will live.

The use of dementia specific tools, protocols, and a formal description of the program which were available for some initiatives were described as supporting a professional approach, but typically not for initiatives characterized by a “*bottom-up approach*” that had originated from enthused and dedicated nursing staff. The wider context of care was recognised as important in supporting professionalism; there was a need to take time for proper care or to take time to get an overview (“*helicopter view*”; Table 5) of the situation. Funding mechanisms such as a lump sum and payment of additional staff were crucial in this respect. The funding of additional specialist services and extra costs were justified by cost savings (in countries where people are often hospitalized) or by a better quality of care and PR around this in other places.

#### Negative representations and stigma

Some identified that there was a dissonance between the representation of good hospice care and supporting people with dementia at the end of life. Negative stereotyping among families and volunteers in hospices, fears that people with dementia unavoidably will disturb others, misguided perceptions of dying, of palliative care, and of nursing homes in some instances hindered the providing of good palliative care or the care deemed best according to the experts. For example, in hospices, staff and volunteers may find that people with dementia “*upset their perfect world*” or “*they disturb the peaceful atmosphere*.” Wandering, loss of decorum, and yelling were particularly problematic in their view, for other patients, families and staff. “*People who ‘re afraid of dementia, and untrained, will fail.*” “*Some may say, end-of-life care for a person with dementia, no; it drives me crazy, I can’t do it, and won’t do*.” But others found “*that it did not differ that much from caring for people without dementia*,” or it was more “*like unknown, unloved*.”

One expert argued against specialist provision and suggested that dying is not a special event, it should be normalized and part of everyday life, and therefore dying people should not be transferred to another place. Other experts however, felt that palliative care was often not understood, that it was perceived as a form of rationing and denial of needed treatment, rather than something that would benefit the last days of the patient’s life. An expert explained that families are worried also because they do not know what to expect: “*after a referral [to palliative care services], family usually anxiously await the meeting with the nurse consultant, but usually after one or more visits they regret that they did not request this service earlier*.” The expert mentioned the importance of taking time to explain to families that palliative care is just about “*looking at the situation through a different lens*.”

A nursing home is generally not a place people want to go. However, in the experience of experts, negative public image and negative perceptions about dementia and about nursing homes, may disappear once people are familiar with it. Hospice has a good public image, although a UK expert commented that it is also being viewed as an “elite place” for selected people.

## Discussion

To prepare for developing a best practice and service development for people in the terminal phase of dementia, we used three sources of information representing mainly expert and experiential knowledge and perceived needs. From the EAPC Delphi study, based on both consensus and evidence [4], we identified the avoiding of treatment that is inappropriate or futile in this phase, and a new or renewed focus on comfort as particularly important. The first was reflected in dilemmas around treatment that emerged from the focus group discussions with dedicated families, which were, however, closely tied to concerns about discontinuity in the families’ relationships with the staff. Experts in palliative care, on the other hand, mentioned concerns of families that palliative care was synonymous with not receiving treatment their relatives need. The experts emphasized the need for a focus on comfort in order to professionalize the care with the right attitude, mix of medical and social care, skills and tools.

The families expressed a profound need for continuity in relationships and communication and support to continue care unchanged as long as possible. Negative perceptions about nursing homes may have explained why families perceived them as not in the best interest for the person with dementia and therefore they preferred terminal care at the place of residence if possible. It was, however, the quality of the relationships and continuity of care that were identified as creating homelike environments as much as the location of care. This is reflected in the themes familiarity and communication. Despite complex support needs they resisted involvement of a large team of unfamiliar (professional) caregivers and preferred to rely on a trusted person, often a professional caregiver they knew well, for advice and support.

Familiarly was also a theme that emerged from the interviews with experts about innovative initiatives to provide end-of-life care in the terminal phase of dementia. Preferred, successful models were those in which a representative of a well-trained team takes time and brings specialized care, and education of staff and family to where people are and still ensure continuity of relationships around the patient. Accessibility and good communication were also important to create bonds and a consensus about the goal of care. These things took time not only because of the establishing of relationships and trust, but also because the experts felt there were misrepresentations about palliative care that had to be addressed before care could be provided. Continuity of care was also identified as important from the EAPC recommendations; in this phase of the dying trajectory there is de facto, no time to re-establish or create continuity in the care once it has been lost.

A final theme, which appeared from the expert interviews was how end-of-life care for people with dementia was represented, and the institutions providing it. There was a remarkable difference in negative views of the nursing home as a provider of terminal care whereas views about hospice were mostly positive except for access to hospice being viewed as overly selective and exclusive, a privilege for few. However, in the experience of experts, negative public image and negative perceptions about dementia and about nursing homes may disappear once people are familiar with them.

Table 6 integrates the evidence from the EAPC Delphi study with the newly collected data about experiences from experts in five countries and experiences, needs and perceptions from family members in the Netherlands. It summarizes what we have learnt so far, what has been achieved, and what are remaining challenges. It shows that terminal care involves special needs of patient and family for continuity in all aspects and that it requires an increased focus on comfort. Beneficial effects can be expected if requirements of staff time, sensitive attitude and skills are met. This calls for management, local and national policy, academics and society to facilitate the basis for good terminal care for people with dementia.

**Table 6:** Service development for terminal care in people with dementia and recommendations inferred from the EAPC dementia white paper, expert interviews and focus groups with family caregivers

Prerequisites/requirements: what we need as a basis for good terminal care in dementia	
− Continuity of all aspects of care. Most important: relational continuity. Also, try not to change environment (physical and social environment) but strengthen/honor the person’s identity − Optimal communication may be promoted by at least one central person (“a linchpin;” whether from outside, consultation services, or a coordinator from within a nursing home) who can analyse the situation and connect people (family and professional caregivers) − Flexibility and open lines for communication between professionals − People around who can take time (nursing staff and volunteers) − Selected staff dedicated to optimize comfort for people dying with dementia − Integrate expertise in dementia and palliative care, in a person as well as within a team. Therefore, need staff training and commitment so that they master both dementia and palliative care approach. At the least, they should have a basic level of understanding.	
Perceived benefits – what has been achieved	
− Good communication, raising sensitive issues, addressing stereotypes and fear, resulted in families being satisfied with choice for the services, in retrospect − Respectful care for both patient and family − Creating a homely environment for people with dementia and their family members − Bringing comfort and good symptom management − Withholding of futile curative care (in some countries, especially so in the Netherlands) − Some development and description of services, protocols and tools for dissemination.	
Challenges – what still needs to be solved or requires ongoing work	
− Bring optimal care to where people are without intruding in familiar relationships − Right balance of social and medical services − Address widespread education needs, especially signaling skills of frontline (nursing) staff − Funding mechanisms, especially for extra staff time or organizing volunteer services − Explain palliative care and combat misperceptions of family and staff, and perhaps the general public, of what palliative care can do − Work on more positive representation of nursing homes − Describe and define best practice in detail such as use of which protocols, tools etc. and research into its effects and the most effective elements − Best practice development as a project which hopefully becomes superfluous in time.	

To achieve patient comfort in its broadest sense, good symptom management, and person-centered care and communication are important. These elements are represented by the two EAPC Delphi study domains found most important by experts [4] and are also consistently seen as attributes of a good death [23]. In a systematic review on needs of people with advanced dementia, Perrar et al. [24] additionally found environmental and supportive needs, relating to more practical needs such as the physical environment [25] and which should not be neglected as a means to bring comfort for people with advanced dementia. Also, an optimal approach for people with dementia more generally combines availability of medical care in the background with a frontline of social and human approach by dedicated nursing staff and volunteers.

Continuity of care was of pivotal importance at the end of life also in other qualitative work, where Flemish cancer patients valued the GP for bringing continuity in relations, information, and management [26]. Qualitative interviews with family who lost a relative with dementia in the UK, found that empathy of nursing staff with the person with dementia was difficult but key in providing emotional comfort to the patient, and that good care prioritizes communication with families and care planning [27]. From our study, it seems important to select dementia care staff dedicated to optimize comfort for people dying with dementia, train them and preferably maintain the same staff members involved at the end of life. This may help bring peace, which is central to care in hospices, also to where people with dementia are.

### Limitations

Our work was limited to family perspectives from a single area in the Netherlands and to participants involved in support groups about caring for people with mostly young-onset dementia. The evaluation of the EAPC recommendations were evaluated for relevance for the terminal phase by only a small group of experts. We also did not perform a new systematic literature review. Nevertheless, the EAPC work provided a robust framework and the experiences of family and professional caregivers, also those who developed new services completely bottom-up from their own perceptions of what people need, helped to better understand how to really serve people with dementia in the terminal phase and their families.

### Recommendations for practice and research

A new service may start from either dementia care, where people are and continuous care is preferred, or from palliative care, which is common in the UK [28]. A mobile palliative team that specializes in palliative care for people with dementia and has the time, authority and expertise to support professional and family caregivers, is a promising model. Compared to transfer to a hospice, it may better meet the needs of families and patients for continuity of care and relationships in addition to appropriate treatments. Such a team may improve care in the Netherlands, where most people die in nursing homes and specialized elderly care physicians on the staff are well equipped to withhold futile medical care. A model with a mobile team could also address the need to support and resource poorly educated nursing staff in nursing homes and in home care to provide holistic care. An outreach nurse can work with available (nursing) staff and also help families, and that model also offers on-the-job learning. We will need to take into account possible rivalries between domains and professionals, and factors that stimulate cooperation between general and specialist palliative care such as good communication as the result of personal liaison between providers, and clear definition of roles and responsibilities [29–34].

A hospice or specialized centre is more visible as a beacon of best practice and would address the principle of equal access for people with dementia, but it is unclear how people would be identified for admission and how this would normalize good practice in terminal care for people with dementia. Further, a unit with a few beds may serve needs of some people staying at home, and perhaps it can prevent a late hospital admission but the often empty, available bed and funding problems should be tackled.

Our work may help in considering setting up services in other countries although the context of what has been achieved and remaining challenges may differ from the situation in the Netherlands. Despite the fact that hospice care for people with dementia was described in US studies around 30 years ago [14, 15], this paper has made explicit the different perspectives on how to (further) develop services for people with the dementia in the last phase. Some belief that a cancer palliative care model equally applies to dementia [35], or wonder if end-of-life care is special at all although it seems more difficult to provide person-centred care if the person cannot express him- or herself well due to advanced dementia or perhaps superimposed acute illness [36]. Others feel we should use the rich sources of knowledge from within long-term care to develop and invest in the expertise of the existing workforce to improve care at the end of life [37]. Our approach with expert interviews was to learn from those who already bridged dementia and palliative care.

The comparison of how different models of palliative care have supported terminal care for people with dementia will help to develop and refine our understanding of what constitutes best practice. This paper has identified a number of challenges to implementation of palliative care specific to people with dementia that include how to involve family carers in decision making, navigate competing accounts of how specialist and generalist services should work together, and more generally, the lack of training and funding, need for culture change, and timely identification of patients for referral to palliative care services [38]. There is a lack of interventions that address the particular challenges that dying with dementia pose and studies may not take into account how culture and context informs how care is being provided [39]. Jones and colleagues highlighted the need to influence priorities and promote multi-disciplinary care in local service organisation to integrate end-of-life care in advanced dementia in the UK, in addition to training and support for both family and professional caregivers [40]. Along with Goodman et al., [8, 39] they highlight the need for a theoretical perspective to developing interventions and services: one that is dementia specific and can accommodate the inevitable uncertainties that arise around who should be involved in decision making, where is the best place to provide care, what resources are available and how to maintain staff awareness and expertise over time. This requires opportunities for reflection and service models that can accommodate or “hold” the competing views and uncertainties, presented in this paper, of what is the best way to provide care. Davies et al. [41] call for more empirical studies to understand what works when integrating nursing home care with health care services; their review included only one study in dementia, which focused on addressing challenging behaviour not necessarily in the terminal phase. Future research is clearly needed to evaluate newly developed services and to test (cost)effectiveness and active ingredients that make the difference, and provide specific recommendations for special groups in addition to general recommendations for planning palliative care services such as targeting disease and prognosis with admission and discharge criteria [42].

## Conclusion

Specific expertise is needed in dementia at the end of life, yet continuity of care should be maintained. A mobile team that specializes in palliative care in dementia and supports professional and family caregivers is a promising model. Compared to transfer to a hospice in the last weeks or days, it has the potential to address the priorities of families and patients for continuity of care, relationships and specialist expertise.
